# Whoosh! visual depictions of direction, speed, and temporality: a corpus analysis of motion events in global comics

**DOI:** 10.1515/mc-2025-0006

**Published:** 2025-09-19

**Authors:** Irmak Hacımusaoğlu, Bruno Cardoso, Neil Cohn

**Affiliations:** Department of Communication and Cognition, Tilburg University, Tilburg, Netherlands

**Keywords:** depicted motion events, path and manner, static motion, visual language theory, visual lexicon

## Abstract

Representing motion in static images is a challenge due to the limitations of two-dimensional visual media. Such motion depictions are common in visual narratives, yet no studies have extensively analyzed their properties. This study examines how lexicalized visual cues contribute to motion events by analyzing 315 comics across three studies. Study 1 focuses on the frequency of motion cues and path segments, revealing that midpoints of paths were depicted more frequently than endpoints or starting points, consistent with prior corpus findings. Study 2 then explores how different cues relate to the explicitness of direction, manner of speed, and temporality. While most cues were associated with explicit paths, others, like partial repetition of postures, were more implicit. Also, backfixing and suppletion lines showed no positive relationship with any kind of paths but related to speed of motion. Cues that rely on indexicality also related to implied moments of action. Finally, Study 3 used Principal Component Analysis to uncover broader patterns in the data, revealing more nuanced groupings of properties of motion events than a binary explicit/implicit distinction. Overall, this study offers new insights into the ways static images convey dynamic information by showing they are systematized in a visual vocabulary.

## 1 General introduction

**Figure 1:**
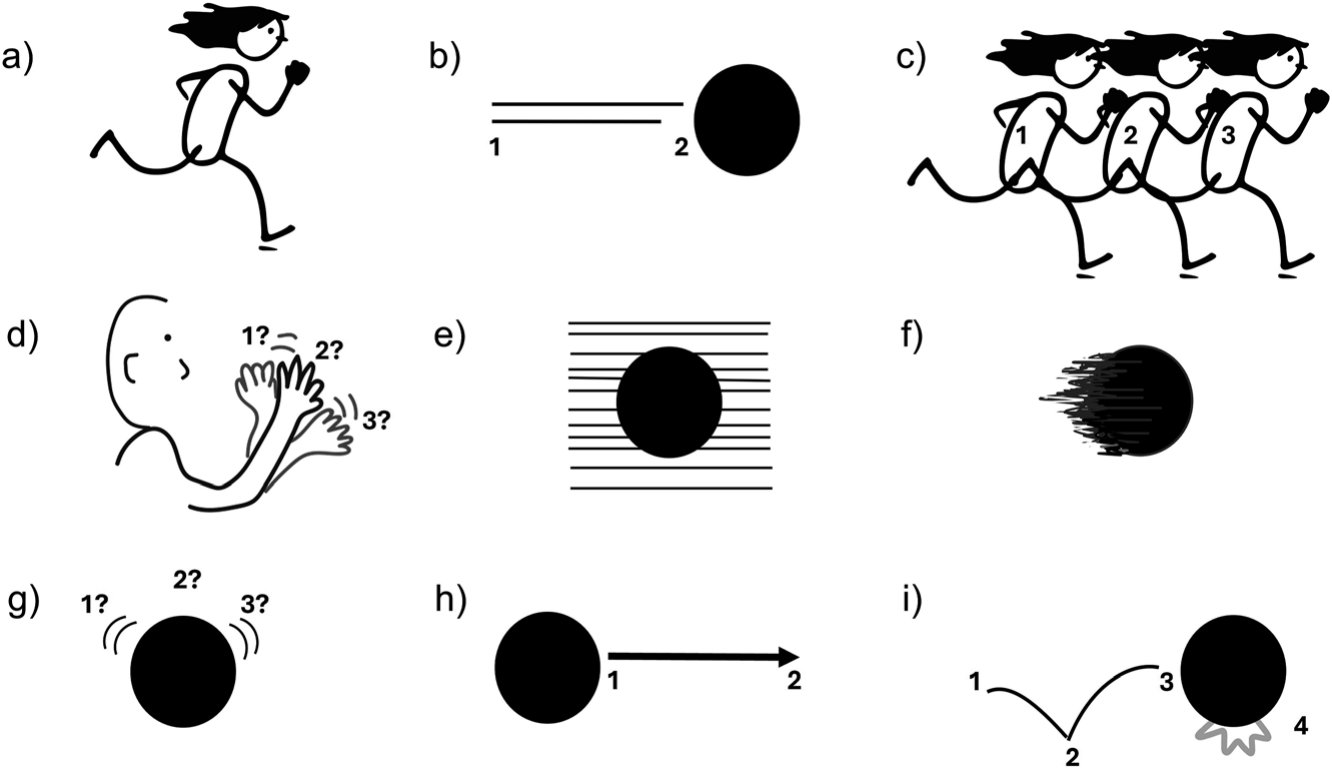
Lexicalized motion cues. (a) Postures, (b) motion lines trailing behind movers, (c) full polymorphism or full repetition of figures in different positions to represent motion, (d) partial polymorphism, (e) backfixing lines set in the background to show speed, (f) suppletion lines replacing parts of the movers to indicate speed, (g) circumfixing lines surrounding movers to indicate vibration, (h) future lines pointing toward a future state, and (i) impact stars showing collision or impact resulting from movement. Numbers correspond to multiple moments implied at once, accompanied by question marks if the exact order remains ambiguous. The running figures were taken from the open-source illustration library, CocoMaterial.

While expressing motion is a fundamental aspect of human communication cutting across modalities (Ünal et al. 2023), representing motion through the visual-graphic modality, such as in static images, is challenging due to the inherent limitations of a two-dimensional medium. Despite this, there are systematic ways to depict motion (Cutting 2002), such as drawing figures’ postures in the middle of an action (Figure 1a) or adding motion lines behind objects to indicate the path they have traveled (Figure 1b). Both postural cues and motion lines have been studied in psychology literature for their role in implying motion in static images (e.g., Friedman and Stevenson 1975; Kawabe and Miura 2006). However, other systematic ways to represent motion in static images, such as those used in comics, have received less empirical attention. Therefore, this work aims to investigate a broader set of motion cues to identify the componential patterns that are systematically used to depict motion. More specifically, through a corpus analysis of comics, here we examine whether certain cues relate to different aspects of motion events.

Motion events – when entities change location or position over time – include components such as path directionality (starting point/source, midpoint/route, or endpoint/goal) and the characteristics or manner of motion (Talmy 2000). Various visual cues draw out these features in static images in different ways. As mentioned, the baseline method is showing figures’ postural cues for actions, which conveys basic manner information like running (Figure 1a). These depicted postures show a snapshot of a single moment instead of pointing to earlier/future moments of action. This means that the full traversal is not represented in the graphics, but the snapshot-nature of postures do help people deduce upcoming action positions (Kawabe and Miura 2006) as seen with static photographs (e.g., Freyd 1983; Kourtzi and Kanwisher 2000). Thus, it is possible to understand the direction through the aid of postural cues (e.g., in Figure 1, the running person is going to the right, not the left).

The other common method, motion lines represent the traversed path by marking both the start (source) and end (goal) points of a path, as the numbers indicate in Figure 1b. Unlike postures, they inherently collapse multiple snapshots into a single representation to show where the object once was and where it currently is. Thus, they not only mark the past location(s) of the mover but also index the direction. The shape of motion lines also varies, contributing to different manner information that may not be captured by postures, such as spinning or bouncing (Hacımusaoğlu and Cohn 2023), or by objects that do not possess postural information.

Motion lines have been studied extensively for how they clarify the direction of motion (Francis and Kim 1999; Kim and Francis 1998), aid motion comprehension (Cohn and Maher 2015; Ito et al. 2010), and convey manner of speed (Carello et al. 1986) with an increase in their numbers (Gillan and Sapp 2005; Hayashi et al. 2012). However, there is little consensus on how these lines derive their meaning (Hacımusaoğlu and Cohn 2023). Some accounts suggested they reflect the signals or motion streaks that disambiguate the direction in visual perception (Burr 2000; Burr and Ross 2002), while others proposed they act as metaphors for actual path marks (Forceville 2011; Kennedy 1982). These accounts treat motion lines as isolated cues, whereas motion lines are better understood as part of a broader set of motion cues.

**Figure 2:**
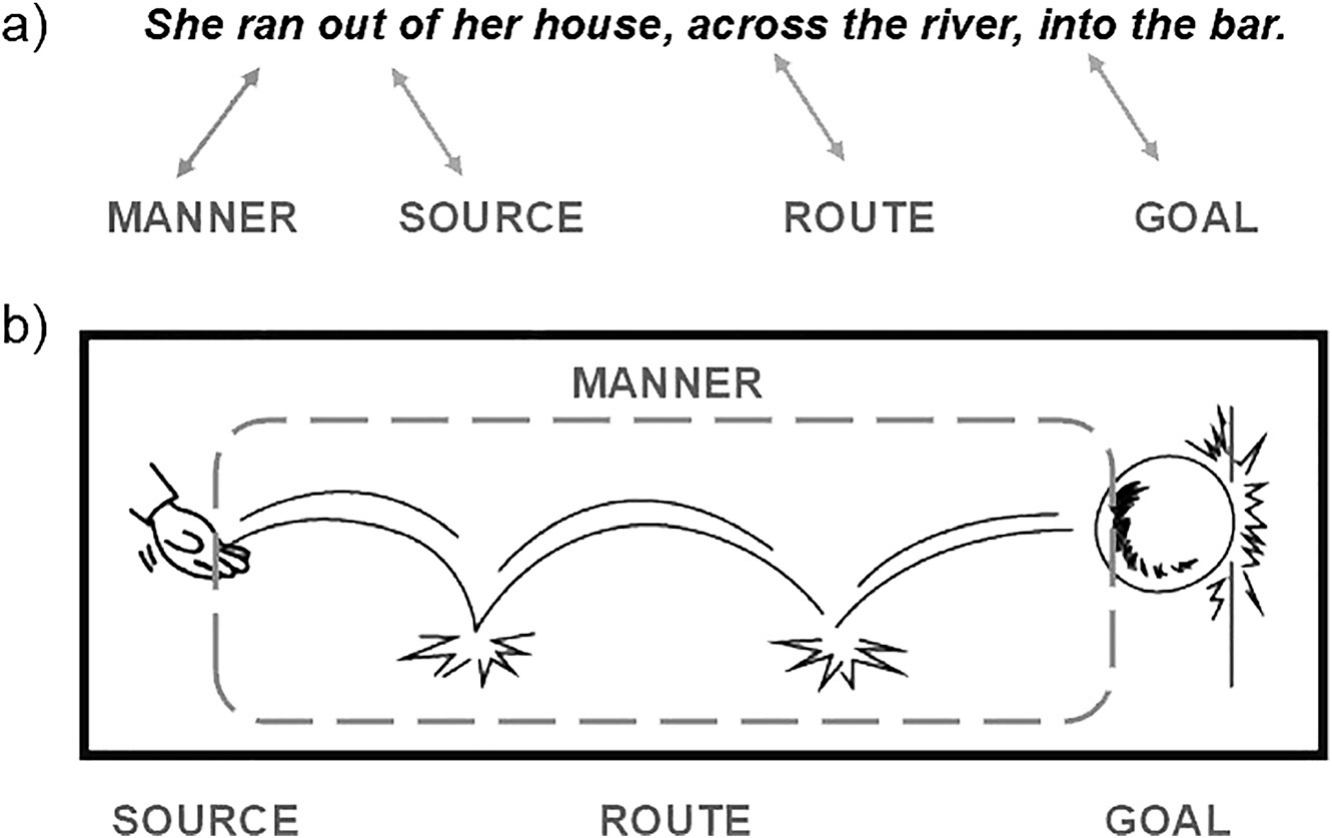
Path and manner information conveyed in verbal and visual-graphic modalities. (a) An example of how some spoken languages encode such information within prepositions and main verbs, respectively. (b) A visually depicted path with the use of motion lines, including all path segments (source, route, and goal). While lines correspond to the full path traversed, their shape conveys the manner of bouncing, and the impact star attached to the ball marks the endpoint.

Indeed, several motion cues or constructions in static images are conventionalized as items in a visual vocabulary. This idea builds upon Visual Language Theory (VLT) (Cohn 2013), which posits that pictures are structured using principles similar to those of spoken or sign languages. According to VLT, people cognitively encode patterned schemas (i.e., items of a visual vocabulary) through exposure. Parallel to spoken and sign languages, these lexical items represent patterned correspondences between form (graphics) and meaning, and use certain combinatorial strategies – such as affixation, repetition, suppletion (Cohn 2018). For example, postures can stand alone morphologically as a meaningful unit while motion lines need to be attached to their stem i.e., a mover, to gain a meaning of movement as a visual affix. Also, the lexical items can use different types of meaning-making such as iconicity i.e., resemblance between the sign and its object as in postures and/or indexicality i.e., when the sign points to its meaning as in motion lines (Peirce 1902). In spoken languages, main verbs or particles can encode motion paths or manner information depending on the linguistic system used (Figure 2a illustrates one method). Likewise, different components of motion can be also encoded in different visual lexical items or parts of a single item (as in Figure 2b). Motion lines are one such encoded lexical item, but several others also highlight different aspects of motion.

Another lexicalized motion cue is *polymorphism* in which postures repeat either fully (Figure 1c) or partially (Figure 1d) to give a sense of movement, which denotes multiple moments at once (indicated by multiple numbers in Figure 1). This cue of full polymorphism, where multiple poses extend an action across a distance, can convey direction, with each pose potentially ordered temporally (see Figure 1c). However, the order might remain ambiguous, like in some partial polymorphism (e.g., the waving hand in Figure 1d) where the repeated poses render the directionality and order of moments less sequentially distinct.

Moreover, some lines deviate from typical motion lines. Parallel straight lines set in the background or *backfixing lines* (Figure 1e) depict the mover in the middle of a path, without showing a past or future moment of action. They also do not inherently show direction because movers appear in front of the lines and thus the motion could be either leftward or rightward. Rather, backfixing lines denote fast speed and they do so from a subjective point i.e., making it seem as if viewers also move as fast as the depicted mover (McCloud 1993).

Lines can also replace parts of movers by using *suppletion lines,* which, like backfixing lines, depict the mover at a midpoint in their path, with fast speed (Figure 1f). Unlike motion lines, they do not show the source of the action nor imply an endpoint, thus the full traversal is not shown or indexed in the graphics. However, these lines can index the direction that the mover heads toward.

Both backfixing and suppletion lines are akin to the streaks or blurs which appear either on the background or on the objects themselves respectively, while capturing a fast-moving stimulus with a camera (Hacımusaoğlu and Cohn 2025). Their shapes typically appear straight, which also tells us these lines do not correspond to the paths themselves, in contrast to the shapes of motion lines, which can indicate varying shapes of the paths depending on different manner of motion (e.g., a curved motion line implies a curved path).

In addition, short lines can surround movers to give a sense of movement (Figure 1g). This cue of *circumfixing lines* remains more implicit in conveying motion as they do not extend across a distance but just imitate the contours of the movers. Similar to partial polymorphism, they convey multiple moments at once by corresponding to multiple states (1, 2, and 3 in Figure 1g) of a mover. When attached to both sides, they indicate back-and-forth movement without specifying the direction and thus without a certain temporal order.

The inverse structure of motion lines appears in *future lines* (Figure 1h), which are arrows attached in front of movers to clarify a path yet to be traversed, thereby indexing the direction toward a future moment instead of past. Thus, they also collapse multiple moments, as numbered in Figure 1. These lines with arrowheads might be more apparent in instruction manuals or guidelines (de Souza and Dyson 2008).

Finally, *impact stars,* a visual affix of a star-shaped burst, marks where the motion left an impact in the past, as in Figure 1i. While impact stars do not show paths directly, they show a resultative state of action and imply a past moment with their indexicality. In that sense, they serve as secondary cues that reinforce the otherwise conveyed action. For example, motion lines depict the path of a bouncing ball, and this can be complemented by impact stars to index where the ball hit the endpoint (see Figure 2b for an example).

To sum up, similar to how path or manner information is encoded in main verbs or particles depending on the linguistic system (Talmy 2000), visual cues also encode such components of motion in different lexicalized patterns (Hacımusaoğlu and Cohn 2023). Our prior corpus work (Hacımusaoğlu and Cohn 2022) analyzed some of these cues and path segments (source, route, and goal; Figure 2) within the Visual Language Research Corpus or VLRC (Cohn et al. 2023) which included 85 comics across East Asia, Europe, and North America annotated with properties of motion events. In that work, we observed that midpoints or routes (see Figure 2b) were depicted more often than sources or goals, and both postures and motion lines were correlated with routes, without being depicted together. In addition, although we did not test this in the corpus, we argued that motion lines show paths more overtly, because they mark the starting/end points of action, while other lines remain more implicit in how they depict paths.

Given these preliminary findings and the features of other cues that remain unexamined, this work aims to replicate prior results in a new corpus and then to explore the structural patterns of depicted motion events. We specifically ask: Do motion cues differ in their relation to explicitness or implicitness of paths (RQ1)? Do higher number of motion lines, backfixing lines, and suppletion lines relate to faster action (RQ2)? Do cues having indexicality like impact stars relate to implied moments of action (RQ3)? Are there any overarching patterns for the clustering of certain motion cues and path segments with explicit or implicit paths (RQ4)?

We address these questions by using the TINTIN Corpus consisting in total of 1,030 annotated comics from 144 countries or territories around the world. Within the TINTIN Corpus, 315 comics had complete annotations for motion events. Along with the cues and path segments annotated in the VLRC, annotation schemes in the TINTIN Corpus also include more fine-grained annotations of visually depicted motion events (e.g., more types of cues, number of motion lines, mover types etc.). Thus, to investigate how motion events manifest in sequential images, all analyses of motion events look at this sub-selection of the TINTIN Corpus.

We begin by establishing whether motion cues and path segments vary in their frequencies in the TINTIN Corpus, as found in the VLRC (Study 1). We then query the relationship between motion cues and paths in more depth based on the research questions we posed above. To do so, we explore the relationships between cues and explicitness of direction, speed, and temporality, respectively (Study 2). Finally, we use an automated dimension reduction method, Principal Component Analysis, to uncover broader patterns of motion events (Study 3).

## 2 General methodology

### 2.1 Materials

**Figure 3:**
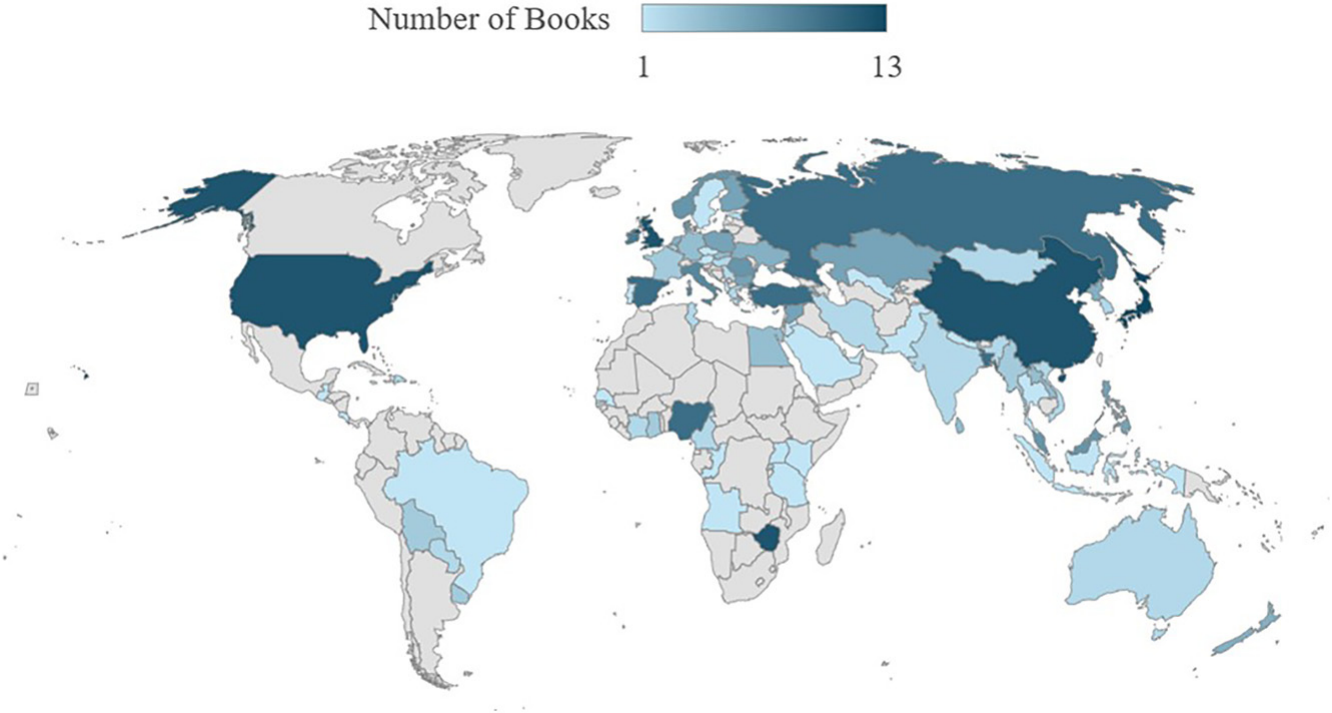
The map shows a gradient of number of books annotated with motion events schemes. The darker the color, the more books come from the marked region.

The complete TINTIN Corpus includes 1,030 comics from 144 countries or territories gathered using convenience sampling with the primary criterion being global coverage (Cohn et al. Under Review). Within the total corpus, 315 comics from 81 countries were fully annotated for motion events (see the map in Figure 3).

### 2.2 Procedure

**Figure 4:**
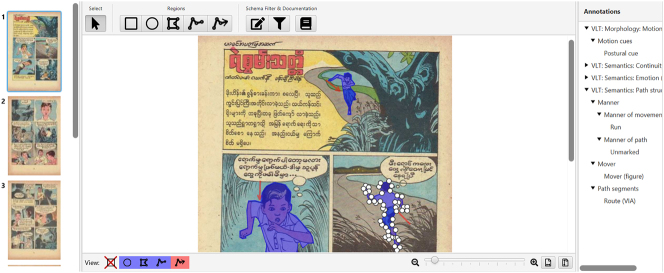
An example of the MAST annotation screen. The bar on the top displays the tools to annotate comics, and the right side of the screen shows the annotations. The comic annotated here is *Courage* © Pangyi U Sein.

Motion events in the TINTIN Corpus were annotated using the Multimodal Annotation Software Tool or MAST (Cardoso and Cohn 2022). MAST provides various tools to define or draw regions around areas of interest (e.g., objects) within an uploaded document, in our case a comic page (Figure 4), or to elaborate on content, such as using the arrow tool to indicate the direction of motion. The coder can then select these regions and annotate them with specific classifications.

The analysis of motion events with MAST was carried out by 8 coders who used the *VLT: Morphology: Motion Events (v.2)* and the *VLT: Semantics: Path Structure (v.2)* annotation schemes (Hacımusaoğlu 2024a, 2024b). Each coder was trained in both schemes and implemented in practice comics until their annotation abilities were sufficient for annotating the actual corpus. Annotations were double-checked and discussed weekly as a group.

The annotation scheme *VLT: Morphology: Motion Events (v.2)* (Hacımusaoğlu 2024a) was used to annotate the motion cues (postures, motion lines, circumfixing lines, backfixing lines, suppletion lines, future lines, impact stars, partial polymorphism) as well as their properties, if applicable (quantity of lines, line shape, and impact star shape). For instance, the number of motion lines was annotated as either a single line, double lines, or several lines. After annotating the morphology of cues, coders used the scheme *VLT: Semantics: Path Structure (v.2)* to annotate the meaning (Hacımusaoğlu 2024b). This scheme included annotations for the mover type (object or figure), path segments (source/starting point, route/midpoint, goal/endpoint), paths (explicit or implicit), manner of action, and physical paths such as tracks left on mud by vehicles. Coders used the arrow tool to indicate direction of motion in case the path being traversed was explicit.

Regions could be annotated with multiple annotations. For instance, if the mover is depicted at a starting point, the region drawn around the mover was annotated with both mover type and the path segment source. However, if the mover was at a midpoint or endpoint, but the starting point of the action is depicted in the scene, then that would be only annotated as the path segment source (see the depiction of the source in Figure 2b without the mover ball). Finally, MAST also enables annotators to add notes when some visual sign was not listed in the annotation scheme, which could be used to specify annotations of moving objects as “vehicles”, or to specify manner as “driving” in cases where movers were depicted as four-wheeled vehicles.

### 2.3 Areas of analyses

The overall areas of analyses were motion cues (*postures, motion lines, circumfixing lines, backfixing lines, suppletion lines, impact stars, future lines*), path segments (*source/starting point, route/midpoint, goal/endpoint*), explicitness of paths (*explicit* or *implicit*), manner of movements (*walk, run, drive*), quantity of motion lines, and mover types (*object* or *figure*). In addition to these annotated variables, we computed a new variable, *implied moments of action*, by looking at whether a path segment is annotated without a mover (like a future goal depicted in the scene that the mover has not reached yet).

The first baseline study sought to establish frequencies of motion cues and path segments averaged per book out of the total number of pages. Then, Study 2 investigated the relationship between cues and paths at the panel level. Specifically, we looked at (1) out of all cues, which ones relate to explicit and implicit paths (2) whether backfixing lines, suppletion lines, and motion lines (double and several lines) relate to manner of walking, running, and driving, and (3) out of all cues, which ones relate to implied (future/past) moments of action. Finally, in Study 3, we searched for overarching patterns in the motion events data by looking at instances of motion cues, path segments, explicitness of paths, implied moments in which a path segment appears without a mover, and mover types at the panel level. The data for all studies are accessible in its DataverseNL repository, https://doi.org/10.34894/ABSVR8.

## 3 Study 1: frequencies of motion cues and path segments in the TINTIN Corpus

Study 1 looked at the frequencies of motion cues and path segments across the books in the TINTIN Corpus. Our prior work using the VLRC indicated there are more postures and motion lines than all the other cues i.e., polymorphism, backfixing lines, suppletion lines and circumfixing lines (Hacımusaoğlu and Cohn 2022). Here, we expand our selection of motion cues to include impact stars and future lines. We expected to replicate findings that postures and motion lines appear the most across comics.

Analysis of the VLRC also showed that routes are depicted more often than goals which were more frequent than sources (Hacımusaoğlu and Cohn 2022). Midpoints are important for conveying motion because they help a viewer to deduce upcoming endpoints. Experimental work has shown that viewers can estimate the upcoming position by looking at the postures of movers in the middle of actions and that holds for motion lines as well (Kawabe and Miura 2006). Thus, postures and motion lines showing routes (Hacımusaoğlu and Cohn 2022) make it possible to comprehend static motion which would otherwise be difficult (Cohn and Maher 2015). We therefore expected to replicate findings from the VLRC that routes are more predominant than other path segments in the TINTIN Corpus.

### 3.1 Methods

#### 3.1.1 Areas of analyses

Here we analyzed motion cues (postures, motion lines, circumfixing lines, backfixing lines, suppletion lines, impact stars, partial polymorphism, future lines) and path segments (source or starting point, route or midpoint, goal, or endpoint).

#### 3.1.2 Data analyses

We summed all instances of each cue or path segment within a book and divided that total by the number of annotated pages in the book. This yielded a mean score for each cue or segment per book, providing the average number of occurrences per page.

For the Analyses of Variance (ANOVAs), we log-transformed the data to reduce skew. We added 1 to the mean values before applying the logarithm to handle cases where the mean was zero since log 0 is undefined. This adjustment ensured that zero values remain zero after transformation (because log 1 is 0), allowing us to include all data points in the analysis. We compared the proportions of motion cues and path segments per page across books using two repeated measures ANOVAs. We applied a Greenhouse-Geisser correction if sphericity was violated and in case of main effects and/or interactions found, we proceeded to Post-hoc analyses with Bonferroni corrections. While reporting, F-statistics and degrees of freedom were rounded to two decimal places.

### 3.2 Results

#### 3.2.1 Motion cues

**Figure 5:**
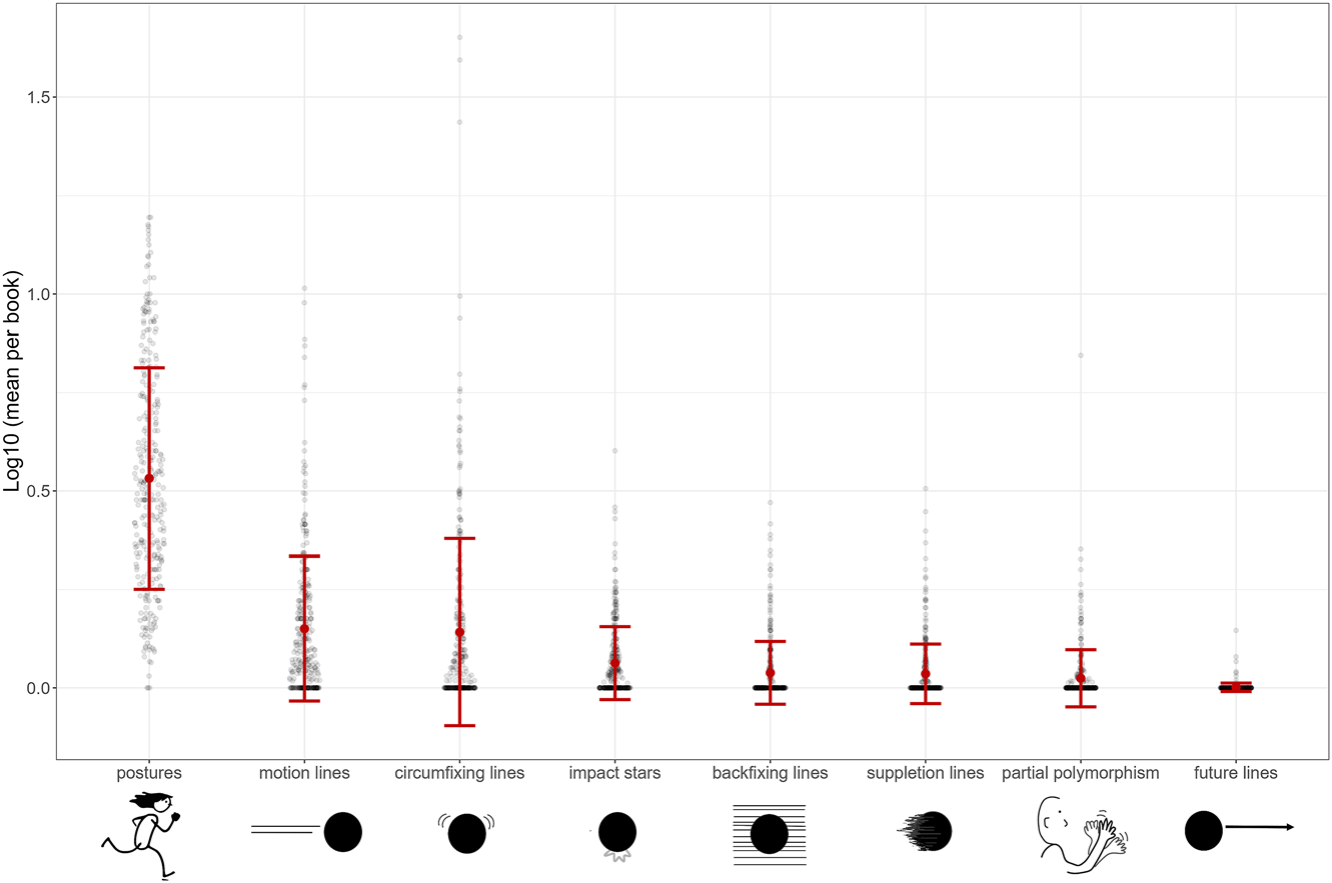
The *Y*-axis shows the log-transformed values, averaged across the total number of pages per book, for each motion cue represented on the *X*-axis. Each gray dot shows a comic. The larger red dots indicate the mean with error bars representing standard deviations.

As shown in Figure 5, the first analysis found differences in the frequency of motion cues (postures, motion lines, circumfixing lines, backfixing lines, suppletion lines, impact stars, partial polymorphism, future lines), *F*(3.08, 967.69) = 473.72 *p* < 0.001, η^2^ = 0.719. Post-hoc analyses indicated postures occurred the most (all *t*s > 33.79, all *p*s < 0.001), followed by motion lines and circumfixing lines which appeared more than all other cues (all *t*s > 6.95, all *p*s < 0.001) but did not differ from each other (*p* = 1.000). Impact stars and backfixing lines appeared in books more often than future lines (all *t*s > 3.25, all *p*s < 0.033) but their frequencies of occurrence did not differ from each other (*p* = 0.805) and were comparable to suppletion lines (all *p*s > 0.44). Impact stars were also depicted more than partial polymorphism, *t* = 3.41, *p* = 0.018.

#### 3.2.2 Path segments

**Figure 6:**
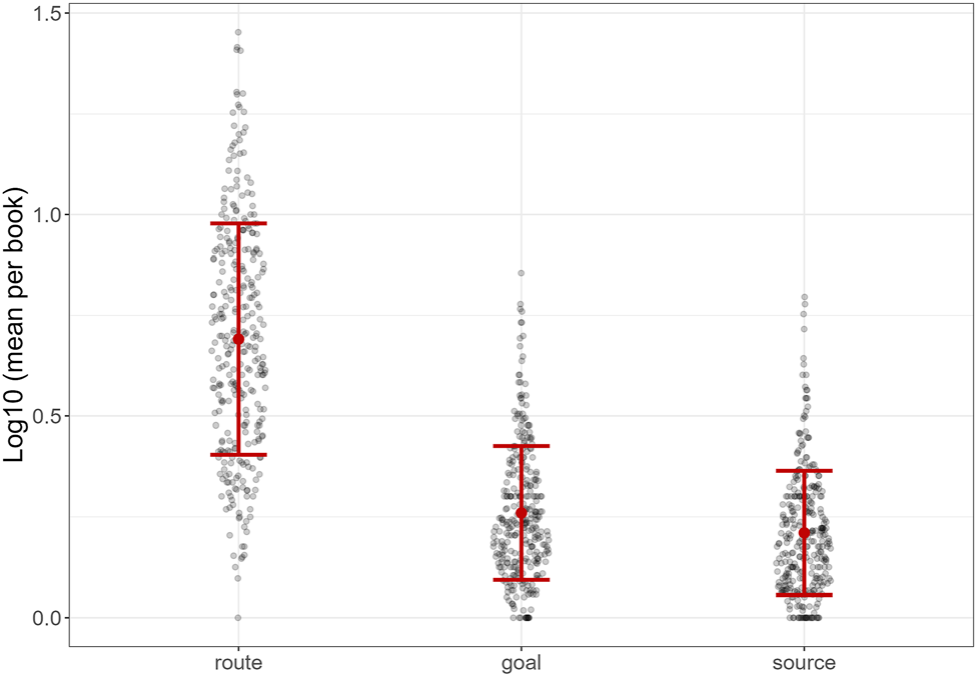
The *Y*-axis shows the log-transformed values, averaged across the total number of pages per book, for each path segment represented on the *X*-axis. Each gray dot shows a comic. The larger red dots demonstrate the mean with error bars representing standard deviations.

Next, as in Figure 6, differences were also found between path segments source, route, and goal, *F*(1.18, 369.79) = 802.14, *p* < 0.001, η^2^ = 0.601. Post-hoc analyses revealed there were more routes than sources (*t* = 36.4, *p* < 0.001) and goals (*t* = 32.66, *p* < 0.001) per book, per page. In turn, goals appeared more often than sources, *t* = 3.74, *p* < 0.001.

### 3.3 Discussion

This study analyzed the frequencies of motion cues (postures, motion lines, circumfixing lines, backfixing lines, suppletion lines, impact stars, partial polymorphism, future lines) and path segments (source, route, and goal) by calculating their average occurrences per page in each book. Overall, we largely replicated prior findings of variations in the proportions of motion cues and path segments.

First, as in Figure 5, postures occurred the most, followed by motion lines and then circumfixing lines. The rest of the cues did not differ from each other, except that there were more impact stars than partial polymorphism. These findings align with our earlier corpus study of the VLRC (Hacımusaoğlu and Cohn 2022). Yet, the addition of future lines and impact stars in the TINTIN Corpus revealed fewer future lines than all other cues, but comparable in proportion to partial polymorphism and suppletion lines. This reduced frequency of future lines may be due to the nature of the corpus, as they may be more peculiar to a visual lexicon specific to instruction manuals or guidelines, which would require a comparative corpus.

The frequencies of path segments also differed (Figure 6). Routes appeared more often than goals, which appeared more than sources, again in line with earlier findings (Hacımusaoğlu and Cohn 2022). This reinforces the notion that routes showing the midpoint of an action might be more essential to convey motion in static images, given that the future position of a mover can be deduced from the postures and motion lines (Kawabe and Miura 2006).

## 4 Study 2: motion cues and path relations: directionality, speed, and temporality

Having established the general tendencies of motion cues and path segments, we next asked about the relationship between motion cues and components of motion such as directionality, speed, and temporality. As introduced earlier, cues might encode different aspects of motion events such as path (e.g., direction) or manner (e.g., speed) of motion, akin to spoken languages (Hacımusaoğlu and Cohn 2023). Some cues directly mark the paths traversed, making it possible to convey the direction more overtly (explicit paths) while others can indicate a sense of movement in which the direction remains ambiguous (implicit paths) (Hacımusaoğlu and Cohn 2022). Along these lines, cues may show a snapshot of movement (e.g., postures) or index past (e.g., motion lines) or future moments (e.g., future lines) of movement. Besides directionality, certain cues are associated with a specific manner information i.e., speed of motion.

**Figure 7:**
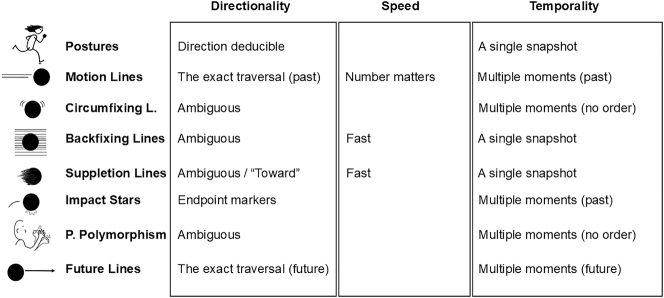
A summary of the features that each cue is suggested to encode based on a lexical account of motion.

These features were discussed for individual cues throughout the introduction, but they are summarized in Figure 7. To further scrutinize these relationships, we turn to the data in the TINTIN Corpus.

We first asked whether motion cues differ in explicitness or implicitness of paths. Psychological experimentation has shown that both motion lines and postures help disambiguate motion direction (e.g., Kawabe and Miura 2006). Also, preliminary analyses of a subset of the TINTIN Corpus has indicated that postures related to both explicit and implicit paths, while motion lines predicted only explicit paths (Degen 2023). However, the remaining cues have not been tested yet, thus our hypotheses for them were built upon the theorized lexicalization patterns alone (Figure 7). Namely, we hypothesized that explicit paths would relate to cues which aid in understanding direction (postures), or which directly index paths traversed (motion lines) or to be traversed (future lines). On the other hand, implicit paths would be related to cues that indicate multiple positions without a specific order (such as partial polymorphism and circumfixing lines), that do not overtly depict paths but instead show movers at a midpoint with fast speed (such as backfixing and suppletion lines), or that mark a point of collision along a path without showing the full traversal (impact stars).

The second question was whether certain cues are associated with fast speed, as suggested by psychological experiments. In experimental work, both suppletion and backfixing lines were rated as indicating faster movement compared to motion lines and bare objects (Hacımusaoğlu and Cohn 2025), a result that manifested in response times for time duration assessments of when participants thought the static object would reach its goal. Additionally, more motion lines resulted in higher speed ratings and shorter time durations, aligning with prior research (Gillan and Sapp 2005; Hayashi et al. 2012). Krajinović et al. (2024) also found more lines were attached to running figures than walking figures in the TINTIN Corpus – again as a primary difference related to speed. Based on these findings, we hypothesized that cues encoding faster movement (backfixing, suppletion lines, and higher numbers of motion lines) would be related to faster actions, such as running or driving, as opposed to slower actions, like walking.

Finally, we also examined how motion cues convey temporality. Given that temporality goes along with the markers of previous or future locations, we hypothesized cues that index past (motion lines and impact stars) or future locations (future lines) of movers would be associated with implied moments of action. In contrast, cues showing a single snapshot in time (postures, backfixing lines, and suppletion lines) would not be linked to past/future moments of movement.

### 4.1 Methods

#### 4.1.1 Areas of analyses

To explore the features of motion cues, we here analyzed whether the paths are explicit (the direction of motion is represented in the depictions or can be understood) or implicit (the direction remains ambiguous). We used annotations of manner of movement as a proxy for speed, since walking and running share similar postures and primarily differ in speed, following the approach taken by Krajinović et al. (2024). In addition to walking and running, we also included driving as a manner of movement in the current study.

**Figure 8:**
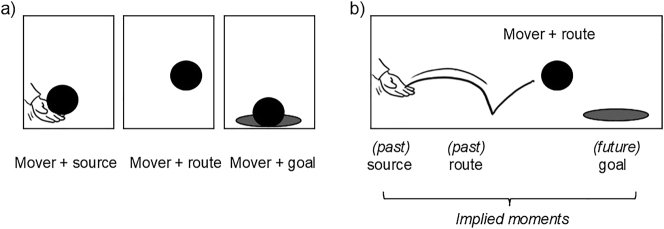
Two illustrations showing where the mover appears at a path segment or where past/future moments along a path are implied: (a) The mover is at the starting point/source, midpoint/route, and endpoint/goal, with the same region annotated as both the mover and the corresponding path segment. (b) Besides the mover at the midpoint of its path, this scene also depicts the starting point already passed, the past route represented by motion lines, and the intended endpoint. Depicted prior or future states not annotated as movers were “implied moments” of action.

Finally, to analyze the temporality of motion cues, we considered regions annotated with path segments but not with movers as *implied moments* of past/future action. As explained in the procedure, if the mover was at the starting point, the same region could be annotated with both mover and source (Figure 8a). If the mover was annotated at the mid/endpoint, then a depicted source would be annotated on a different region (Figure 8b). We classified the latter case as an implied moment. Another example of implied moments could be motion lines showing where the object once was. Lines themselves were annotated without the mover as they show a past moment. Finally, if there was a depicted goal but the mover was not there *yet*, this counted as an implied moment of future (Figure 8b).

#### 4.1.2 Data analyses

To assess the relationships between cues and directionality, speed, and temporality, respectively, counts of each instance per panel were used in Generalized Linear Mixed Models (GLMM). Each model was specified using the Poisson family with a log link function, appropriate for the count data. Books were used as random effect factors in all analyses to account for the nested data structure.

The first model aimed to investigate directionality, where counts of explicit paths were the dependent variable and cue type (postures, motion lines, circumfixing lines, backfixing lines, suppletion lines, impact stars, partial polymorphism, future lines) were fixed effects. Then, the same analysis was conducted with implicit paths being the dependent variable.

Next, to examine speed, we focused on whether different manners (walking, running, driving) were related to specific speed cues (i.e., motion lines – only two or several, backfixing lines, and suppletion lines). To do so, we ran three GLMMs: each model included counts of speed cues as fixed effects and counts of walking, running, and driving were dependent variables, respectively.

For the final feature, temporality, we again added counts of all cues as fixed effects and counts of implied moments as the dependent variable to the model.

### 4.2 Results

#### 4.2.1 Directionality

We began our analyses by looking at the relationship between directionality – its explicitness or implicitness – and motion cues. The first GLMM investigating which motion cues relate to the occurrences of explicit paths demonstrated significant effects of all cues (all *p*s < 0.015), except for backfixing (*p* = 0.213) and suppletion lines (*p* = 0.141). As in Table 1, fixed effect estimates further revealed that postures, motion lines, circumfixing lines, impact stars and future lines were positively associated with the explicit paths at the panel level. Among all significant relationships, only the one between partial polymorphism and explicit paths was negative (*t* = −5.8, *p* < 0.001). That is to say, panels with partial polymorphism were less likely to depict explicit paths.

**Table 1:** Fixed effect estimates for motion cues in the GLMM analysis. The table includes estimates, standard errors (SE), t-values, and p-values for each cue, and asterisks (*) highlight their statistical significance in relation to the explicit paths.

Term	Estimate	SE	t	p-Value
Intercept	−0.745	0.031	−23.790	<0.001
Postures	0.391	0.007	55.971	<0.001*
Motion lines	0.135	0.011	12.485	<0.001*
Circumfixing lines	0.033	0.010	3.338	<0.001*
Backfixing lines	0.060	0.048	1.259	0.208
Suppletion lines	0.049	0.033	1.494	0.135
Impact stars	0.104	0.025	4.201	<0.001*
P. Polymorphism	−0.109	0.019	−5.802	<0.001*
Future lines	0.563	0.209	2.686	0.007*

The second model looking at which motion cues relate to the occurrences of implicit paths in comic panels found significant effects of postural cues, motion lines, circumfixing lines, backfixing lines (all *p*s < 0.024) and almost partial polymorphism (*p* = 0.058). Fixed effect estimates (Table 2) revealed panels with postural cues and circumfixing lines were likely to have implicit paths (all *t*s > 2.02, *p*s < 0.02). In contrast, motion lines (*t* = −5.33, *p* < 0.001) and backfixing lines (*t* = −2.29, *p* = 0.022) were negatively associated with implicit paths in a comic panel.

**Table 2:** Fixed effect estimates for motion cues in the GLMM analysis. The table includes estimates, standard errors (SE), t-values, and p-values for each cue, and asterisks (*) highlight their statistical significance in relation to the implicit paths.

Term	Estimate	SE	t	p-Value
Intercept	−0.751	0.041	−18.381	<0.001
Postures	0.068	0.011	5.938	<0.001*
Motion lines	−0.139	0.026	−5.332	<0.001*
Circumfixing lines	0.031	0.013	2.351	0.019*
Backfixing lines	−0.154	0.067	−2.295	0.022*
Suppletion lines	−0.104	0.063	−1.661	0.097
Impact stars	−0.073	0.043	−1.680	0.093
P. Polymorphism	0.084	0.041	2.028	0.043*
Future lines	−0.542	0.475	−1.140	0.254

#### 4.2.2 Speed

To unravel the relationship between cues and speed, our models targeted the relationship between cues conveying speed (double and several motion lines, backfixing lines, and suppletion lines) and manner of movements with varying level of speeds. The model with manner of walking found significant effects of all cues (all *p*s < 0.001). However, fixed effect estimates (Table 3) demonstrated all relationships found were negative (all *t*s < −3.8, all *p*s < 0.001). In other words, panels depicting walking tend to have fewer motion lines (either type), backfixing lines, and suppletion lines.

**Table 3:** Fixed effect estimates for selected speed cues in the GLMM analysis. The table includes estimates, standard errors (SE), t-values, and p-values for motion lines (double and several), backfixing lines, and suppletion lines. Asterisks (*) highlight their statistical significance in relation to the depictions of manner of walking.

	Estimate	SE	t	p-Value
Intercept	−1.838	0.054	−34.055	<0.001
Double motion lines	−0.606	0.138	−4.394	<0.001*
Several motion lines	−1.115	0.156	−7.130	<0.001*
Backfixing lines	−2.694	0.374	−7.195	<0.001*
Suppletion lines	−1.369	0.360	−3.803	<0.001*

The second model with manner of running also found significant effects of all cues in the model (all ps < 0.043). Fixed effect estimates (Table 4) revealed that these relationships were all positive (all ts > 2.15, all ps > 0.032). That is to say, panels with depiction of running figures are likely to contain these specific speed cues.

**Table 4:** Fixed effect estimates for selected speed cues in the GLMM analysis. The table includes estimates, standard errors (SE), t-values, and p-values for each speed cue, and asterisks (*) highlight their statistical significance in relation to the depictions of manner of running.

	Estimate	SE	t	p-Value
Intercept	−3.437	0.107	−32.191	<0.001
Double motion lines	0.161	0.075	2.161	0.031*
Several motion lines	0.295	0.070	4.216	<0.001*
Backfixing lines	1.051	0.097	10.817	<0.001*
Suppletion lines	0.434	0.072	6.008	<0.001*

The final model with manner of driving found significant effects of backfixing lines, suppletion lines and several motion lines (all ps < 0.049), but not double lines (p = 0.455). As in Table 5, fixed effect estimates for significant relationships were all positive (all ts > 2.31, all ps < 0.035), reflecting that panels depicting the manner of driving were likely to have backfixing lines, suppletion lines and several motion lines as well.

**Table 5:** Fixed effect estimates for selected speed cues in the GLMM analysis. The table includes estimates, standard errors (SE), t-values, and p-values for each speed cue, and asterisks (*) highlight their statistical significance in relation to the depictions of manner of driving.

	Estimate	SE	t	p-Value
Intercept	−10.469	0.864	−12.111	<0.001
Double motion lines	−0.167	0.232	−0.717	0.473
Several motion lines	0.403	0.174	2.320	0.020*
Backfixing lines	1.009	0.234	4.315	<0.001*
Suppletion lines	0.438	0.206	2.123	0.034*

#### 4.2.3 Temporality

As mentioned, cues vary whether they show a single moment in time or index future or past moments of action. Thus, we next delved into the relationship between implied moments and cues. This model with implied moments showed a significant effect of all cues (all *p*s < 0.044) except for postural cues (*p* = 0.814) and backfixing lines (*p* = 0.507). As listed in Table 6, all relationships found were positive (all *t*s > 2.3, all *p*s < 0.022), except for the one between suppletion lines and implied moments (*t* = −6.66, *p* < 0.001). This finding suggests that panels depicting suppletion lines were less likely to have implied moments of action.

**Table 6:** Fixed effect estimates for motion cues in the GLMM analysis. The table includes estimates, standard errors (SE), t-values, and p-values for each cue, and asterisks (*) highlight their statistical significance in relation to the implied moments.

Term	Estimate	SE	t	p-Value
Intercept	−1.746	0.059	−29.430	<0.001
Postures	−0.004	0.017	−0.226	0.821
Motion lines	0.428	0.012	34.291	<0.001*
Circumfixing lines	0.038	0.016	2.421	0.015*
Backfixing lines	0.048	0.070	0.685	0.493
Suppletion lines	−0.305	0.046	−6.660	<0.001*
Impact stars	0.086	0.026	3.314	<0.001*
P. Polymorphism	0.279	0.043	6.553	<0.001*
Future lines	0.595	0.257	2.317	0.021*

### 4.3 Discussion

**Figure 9:**
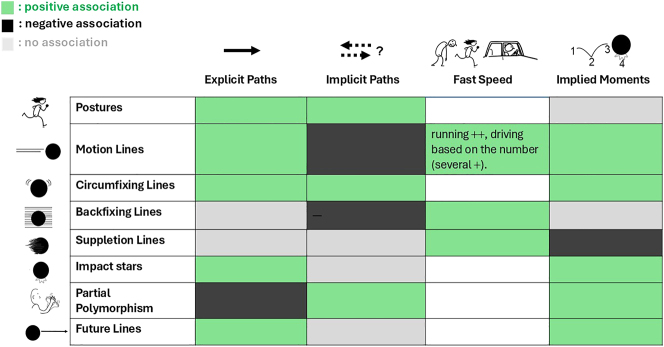
A summary of the statistical analyses with positive (marked with the color green) and negative (marked with the color black) associations found between each cue and features of explicitness or implicitness of the direction, fast speed, and implied moments of action. The color gray indicates no association was found, while uncolored cells denote that no test was conducted.

This section explored the variance in motion cues, specifically their relationship to path directionality, speed, and temporality. The results indicated that cues varied in their association with explicitness or implicitness of directionality. Also, backfixing and suppletion lines were associated with faster action, similar to the higher number of motion lines. Finally, we also observed variation in cues’ relationships to implied moments. Figure 9 summarizes these findings and specifics of each are discussed below.

We began our analyses by looking at cues’ relationship to explicitness/implicitness of paths. The findings were largely in accordance with the hypothesized patterns. First, both postures and motion lines were related to explicit paths at the panel level, in accordance with experimental findings (Kawabe and Miura 2006). Postures were also linked to implicit paths, following preliminary corpus findings (Degen 2023). Also, a positive relationship appeared between future lines and explicit paths, as expected. Meanwhile, partial polymorphism showed a negative association with explicit paths but positive with implicit paths, aligning with its ambiguous directionality.

We also observed unexpected patterns. For example, impact stars yielded a positive relationship with explicit paths despite not directly depicting a path. As discussed, impact stars serve as a secondary cue to strengthen otherwise depicted movement. Because they reinforce primary motion cues like postures or motion lines which can disambiguate the direction, this might have led to an association with explicit paths. Also surprisingly, circumfixing lines showed a positive association with both explicit and implicit paths. We had argued these lines convey a sense of shaking without specifying the direction. However, circumfixing lines sometimes appear one-sided, behind the movers (referred to as contour traces) and then can index where the mover faces. Therefore, this association with both types of paths might stem from annotations not distinguishing between single- and double-sided circumfixing lines. Future annotations might consider examining this distinction.

Regarding speed, we argued that backfixing lines, suppletion lines, and the number of motion lines encode fast speed. Our results indicated backfixing and suppletion lines were likely to appear together with faster action, running and driving, in comic panels. The opposite was the case for the slowest action we investigated, walking. Also, the number of motion lines mattered in terms of the speed of motion they represent as we observed several lines related to the fastest action here driving, but not double lines. This finding supports that the number of lines encode the speed, supporting experimental (Hacımusaoğlu and Cohn 2025) and other corpus findings (Krajinović et al. 2024).

Finally, we turned to cues’ relationship to implied moments based on their indexical nature. In line with the theorized lexicalization patterns, cues indeed differed in their association with temporality. As expected, cues indexing travelled paths (motion lines) or future paths (future lines) appeared in panels together with implied moments of action, as did impact stars marking the impact left in the past. In addition, panels with circumfixing lines and partial polymorphism tended to have implied moments. This was not hypothesized but might be arisen from cues’ multi-snapshot nature (Figure 1d and e), even if these cues do not specify the order of those moments as future/past (Figure 7). Moreover, panels with suppletion lines were less likely to have implied moments of action, going along with the notion that suppletion lines do not mark where the previous place was, or an upcoming place would be.

In conclusion, motion cues differed in the ways they relate to visually depicted motion events such as directionality, speed, and temporality (as summarized in Figure 9). Yet, some cues showed no positive relationship to either implicit or explicit paths, such as suppletion and backfixing lines. These findings raised further questions about whether a binary classification is sufficient to explain the depiction of paths. Thus, we proceeded into an exploratory dimension reduction analysis to further explore the underlying components of visual motion events in our corpus.

## 5 Study 3: Principal components of visual depictions of motion events

We have so far looked at the basic overview of some properties of motion events (motion cues and paths) in the TINTIN Corpus and tested the relationship between them. Building on these analyses, which examined individual cues and their relationships in isolation, our focus now shifts to identifying general patterns of depicted motion events within our corpus data, without predefining any relationships. Are there general properties of motion events that can be revealed through this corpus data? To test this question, we employed a dimension reduction method, Principal Component Analysis (PCA) and explored the underlying structure of visual depictions of motion events. PCA results in new variables called principal components (PCs) which are linear combinations of the original variables. PCs are independent of each other and ordered by the amount of variation they capture in the present dataset, with the first PC explaining the most variation, the second PC explaining the second most, and so on (see a review by Black et al. 2022). This approach has been used in corpus linguistics to examine topics like the covariance of sound elements (e.g., Brand et al. 2021) and in the study of comics to classify page layouts (Bateman et al. 2021).

A key advantage of this approach is its ability to uncover latent dimensions of variation without the need to pre-specify the dimensions of comparison, as emphasized by Bateman et al. (2021). This method would allow us to uncover properties and relationships within the data that may not be apparent through manual inspection alone. Through this automated analysis, we seek to identify commonalities and distinctions in the representation of motion cues and paths and uncover the broader patterns underlying depicted motion. Overall, this approach offers a complementary perspective to analyses we have conducted in the earlier sections, by aiming to identify whether overarching patterns and groupings would arise.

### 5.1 Methods

#### 5.1.1 Areas of analyses

In this study, we explore the potential groupings of movers (object or figure), motion cues (postures, motion lines, circumfixing lines, backfixing lines, suppletion lines, impact stars, partial polymorphism, future lines), path segments (source, route, goal), the explicitness of paths (explicit or implicit), and implied moments.

#### 5.1.2 Data analyses

We calculated the total number of each instance of interest per comic panel. We used JASP version 0.19.1.0 (JASP 2024) where the number of components was decided automatically based on the parallel analysis of principal components. The rotation method was set as orthogonal, varimax and the data was decomposed based on correlations to explore the co-occurrences of different variables in the data.

### 5.2 Results

#### 5.2.1 Principal components

The model resulted in five principal components (PC), χ^2^(40) = 37,211.19, p < 0.001. After the rotation, the first PC accounted for 18.4 % of the total variance, which was followed by PC2 (17.1 %), PC3 (10.7 %), PC4 (8 %) and PC5 (7.7 %). The total variance explained by the model was 61.9 %.

**Figure 10:**
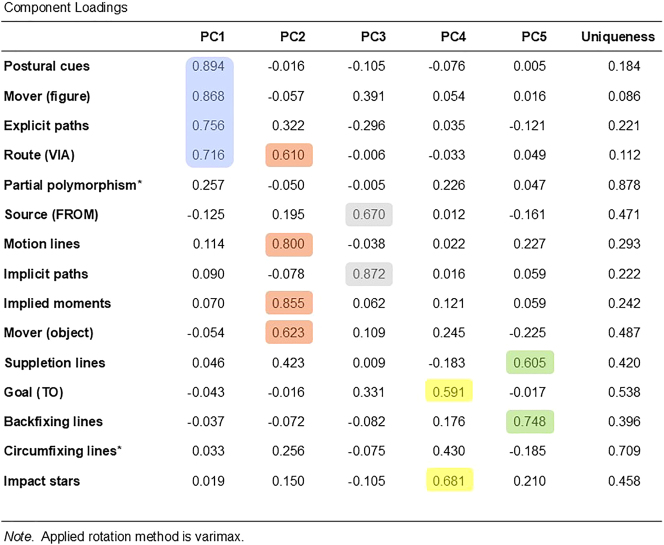
The component loadings of each variable on the five principal components derived from the PCA. The first column lists the variables, and the cells with color represent loadings that exceeded the threshold of 0.5 (lilac for PC1, orange for PC2, gray for PC3, yellow for PC4, and green for PC5). Unique variables that are not associated with any component due to not meeting the threshold are marked with asterisks.

**Figure 11:**
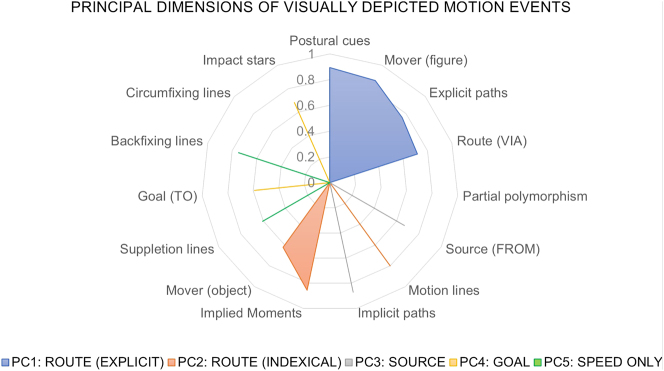
A radar chart showing the principal dimensions of visually depicted motion events in the TINTIN Corpus.

We applied a threshold of 0.5 based on the cumulative contribution of variables with the highest loadings to each PC to prevent the interpretation of too small loadings, as suggested by Brand et al. (2021). Based on this cut-off point, as marked with different colors in Figure 10, PC1 included postural cues, mover (figure), explicit paths, and routes. PC2 consisted of routes, implied moments, motion lines, and mover (object). PC3 included implicit paths and sources and PC4 included impact stars and goals. The final component, PC5 included suppletion and backfixing lines. Circumfixing lines and partial polymorphism were not associated with any component because their loadings did not exceed the cut-off value. Figure 11 shows the components after the threshold has been applied.

#### 5.2.2 Interpretation of components

The calculation of PCs are automatically done based on the data but the components identified by this analysis need to be further interpreted since it is primarily a descriptive approach. Thus, we now delve into the interpreation of each component.

##### 5.2.2.1 PC1: explicit routes

The variables **postural cues**, **mover (figure)**, **explicit paths**, and **routes** loaded onto the first principal component (PC1). In previous work, postural cues have been found to correlate with routes by depicting figures in the midst of paths (Hacımusaoğlu and Cohn 2022), while in Study 2, we observed figures’ postures are related to explicit paths, in line with how they allow readers to anticipate the upcoming position (Kawabe and Miura 2006). Altogether, PC1 can be interpreted as representing *Explicit routes*, where the direction of motion is directly shown in the graphic representation based on the resemblance between moving figures’ depicted poses and their corresponding meanings.

##### 5.2.2.2 PC2: indexical routes

Like PC1, PC2 also involved routes. In this component though, **routes** grouped with **implied moments, motion lines, and mover (object)**. In Study 2, motion lines were related to implied moments of action. Previous corpus works on motion events have also shown that motion lines correlate with routes (VLRC: Hacımusaoğlu and Cohn 2022) and are used more often for moving objects than for figures (TINTIN Corpus: Krajinović et al. 2025). Because inanimate objects are not expected to move autonomously and do not have inherent postural cues to indicate motion or direction, they require extra cues to indicate their movement, such as motion lines clarifying the path traversed through their indexicality. Also, this component linked to neither explicit nor implicit paths. Thus, the second component can be classified as *indexical routes*, which cut across explicit and implicit paths.

##### 5.2.2.3 PC3: sources

The third principal component included **sources** and **implicit paths**. Despite being the least frequently depicted path segment (VLRC and Study 2 here), sources do appear in comics. A key aspect of visually depicted sources is that the path remains mostly implicit. This occurs because the direction is not known at the starting point of the path, and without subsequent action depicted in the same panel, the reader cannot determine the direction of movement. Therefore, this component can be designated as *Sources*, characterized by the implicitness of directionality.

##### 5.2.2.4 PC4: goals

PC4 consisted of goals – the last path segment – and the cue of impact stars. As discussed earlier, impact stars represent the collision resulting from an action, such as depicting a fist reaching another person in a boxing scene or a ball hitting a wall. Thus, they have been argued to mark endpoints, and here they grouped together with goals. In that sense, this component can be categorized as *Goals*, with impact stars serving as their markers.

##### 5.2.2.5 PC5: speed only

The final PC involved backfixing and suppletion lines, without any associated path segments. As mentioned, these two cues do not indicate where the action starts or ends but rather suggest fast speed in the middle of an action. It is important to note that suppletion lines also have indexicality and can contribute to directionality to some extent. However, this component primarily captured their function in denoting speed, grouping them with backfixing lines. Both cues’ effectiveness in depicting speed has been confirmed by experimental work (Hacımusaoğlu and Cohn 2025), and we also observed that they were positively related to faster movements but not to paths in Study 2. Thus, PC5 can be classified as the Speed-only component.

### 5.3 Discussion

In the final study, we sought to understand broader patterns in the representation of motion within static images by investigating whether certain cues, path segments, and implied moments align with explicit or implicit paths of different mover types. An automated analysis of the data resulted in five dimensions: Explicit routes, Indexical routes, Sources, Goals, and the Speed-only component. We further discuss the implications of this analysis below.

We previously argued that paths are divided into two as explicit and implicit paths. Explicit paths were conceptualized as where the direction is understood either by looking at the cues or directly shown in the graphic representation as in postures. Implicit paths were when the traversal of the movers is not shown or occurs off-panel, and the direction remains ambiguous. However, not every cue was associated with either of these paths in Study 2, suggesting this binary classification may not be sufficient to capture the nuances of visual depictions of motion events. PCA provided more nuanced ways of how paths and motion cues aligned.

First, our analysis split routes into two different groupings: As in PC1, representation of action through figures’ postures encodes the direction, and it becomes explicit. As in PC2, routes can be also conveyed through motion lines that index past moments of action, especially to depict movement of objects that lack any postural cue to suggest motion. We had argued motion lines explicitly disambiguate the motion direction (Hacımusaoğlu and Cohn 2022), and Study 2 showed they related to explicit paths. Yet, in this analysis explicitness only aligned with postures. This may suggest even though direction can be understood by motion lines, they do not show direction through iconicity or represent the mover in action, unlike postures. Thus, PC1 and PC2 together suggest differentiation of explicit paths between the direct graphical representation and indexed meaning. Indexical paths or routes may therefore be another class besides explicit and implicit paths, i.e., mostly depicting objects in the middle of a path.

Then, each of other two path segments, sources and goals, formed their own groupings. Sources clustered with implicit paths, in line with how the direction of motion cannot be understood if the rest of the action has not been depicted yet (see Figure 8a, the first panel). The other side is goals – when the action has already been completed. Thus, they made a grouping with impact stars, which strengthen results of an action and thus often mark endpoints. Altogether, so far each path segment made its own grouping while routes, the most prominent segment of depicted paths (Hacımusaoğlu and Cohn 2022) as confirmed in Study 1, resulted in two different groupings based on the mover type.

Finally, backfixing and suppletion lines did not cluster with any path segment, just as they did not relate to either type of path in Study 2. Indeed, these cues remain unique in the ways they depict motion: like motion lines, both are visual affixes to indicate movement, but unlike motion lines, they do not correspond to the path itself. Instead of marking starting or endpoints, they denote the speed of action, as found in our experimental work (Hacımusaoğlu and Cohn 2025) and Study 2 here. These findings again suggest they rather depict fast speed, and this component captured a more nuanced pattern of static motion.

## 6 General discussion

This study examined motion cues and path segments (sources, routes, and goals) of visually depicted motion events in 315 comics from across the globe. Study 1 confirmed that midpoints (routes) were depicted more often than both starting (sources) and endpoints (goals) of actions. Then, Study 2 showed that motion cues differ in their relation to path directionality, speed, and temporality. Finally, a principal components analysis of depicted motion (Study 3) revealed that visual paths may go beyond a merely explicit or implicit classification.

Study 1 confirmed earlier corpus findings (85 comics) about the frequencies of motion cues and path segments (Hacımusaoğlu and Cohn 2022). To summarize, routes were depicted more often than goals, which were more frequent than sources. This suggests that sequential images have structured patterns for depicting movers mostly in the middle of paths, whether through their poses or other motion cues. At the same time, these patterns may also be shaped by broader cognitive mechanisms, such as that endpoints are more focused and remembered than starting points, a constraint which appears in attention, memory, and spoken languages (Papafragou 2010; Regier and Zheng 2007).

In addition, Study 2 revealed that motion cues vary in conveying different aspects of motion. While most cues were related to the explicitness of the direction, partial polymorphism was linked to implicit paths, consistent with how this cue does not overtly indicate the direction. Also, circumfixing lines and postures were associated with both explicit and implicit paths, suggesting that the relative position of circumfixing lines to the mover and the action types of postures may determine how these cues encode directionality. However, neither backfixing nor suppletion lines showed a relationship with explicit or implicit paths, and if they do, it was a negative relationship. This motivated us to conduct Study 3, discussed in detail shortly.

Besides directionality, speed of motion was also systematically related to certain cues. Panels of walking figures were negatively related to any speed cue (motion lines, backfixing lines and suppletion lines), while those cues were likely to appear together with the depictions of running and driving. These results strengthen other findings on backfixing/suppletion lines and fast speed (Hacımusaoğlu and Cohn 2025). Here we similarly show a relationship between increased number of motion lines and faster speed, as running was related to both double and several lines but the fastest action, driving, had a relationship with only several lines. Study 2 also revealed insights about temporality inherent to motion events. As argued, cues indexing a past moment of action (e.g., motion lines, impact stars, future lines) or repeating multiple poses appeared together with implied moments, confirming that it is possible to convey multiple moments at once in static images. Altogether, Study 2 indicated that different cues encode distinctive features of motion events.

Finally, in Study 3 we applied an automated analysis to search for broader patterns in motion events data. This analysis revealed five underlying dimensions of motion events data, dividing each path segment (source, route, and goal) into separate components, and classifying a speed-only component with backfixing and suppletion lines with no associated path segments, as a manner-only construction. Moreover, these dimensions involved two distinct types of routes. More specifically, routes can be explicit for figures through the aid of their postures, while they can be indexical through motion lines for objects that require extra cues to clarify the path they travel.

This differentiation of routes, based on the types of movers, aligns with the idea that inanimate objects lacking any postural cue to suggest movement are not expected to move autonomously. As a result, objects require additional morphological markings, such as motion lines, i.e., a “Mark the unexpected” principle (Krajinović et al. 2025). This finding could further explain why postures and motion lines do not tend to occur together in comics (Hacımusaoğlu and Cohn 2022; Juricevic 2018) despite both being associated with routes (Hacımusaoğlu and Cohn 2022). Although the lack of co-occurrence had been explained as a mismatch between “metaphoric” motion lines and “literal” postural cues (Juricevic 2018), our findings suggest they can both indicate action in the middle of a path, and their use might rather be motivated by the type of the mover and corresponding morphological principles. Altogether, Study 3 offered a more integrative classification of semantical features of depicted motion events.

In conclusion, despite the constraints of a two-dimensional medium, it is possible to depict motion and temporal elements in individual images within a sequence, similar to other modalities. These insights go beyond the well-studied cues of postures or motion lines to suggest different strategies or cues contribute to varying aspects of depicted motion, whether it is direction or speed. Thus, this work sheds light on whether motion lines are merely perceptual, are metaphorical extensions of natural path marks, or are motion cues as a part of a broader visual lexicon. Our findings here support the lexical account, as it reinforces that a range of semantic features manifest in different morphological cues, not a lack of structure (perceptual) or a global metaphor about motion (Hacımusaoğlu and Cohn 2023).
